# Advances in the Management of Primary Membranous Nephropathy and Rituximab-Refractory Membranous Nephropathy

**DOI:** 10.3389/fimmu.2022.859419

**Published:** 2022-05-04

**Authors:** Maxime Teisseyre, Marion Cremoni, Sonia Boyer-Suavet, Caroline Ruetsch, Daisy Graça, Vincent L. M. Esnault, Vesna Brglez, Barbara Seitz-Polski

**Affiliations:** ^1^ Centre de Référence Maladies Rares Syndrome Néphrotique Idiopathique, CHU de Nice, Université Côte d’Azur, Nice, France; ^2^ Unité de Recherche Clinique de la Côte d’Azur (UR2CA), Université Côte d’Azur, Nice, France; ^3^ Laboratoire d’Immunologie, CHU de Nice, Université Côte d’Azur, Nice, France; ^4^ Service de Néphrologie-Dialyse-Transplantation, CHU de Nice, Université Côte d’Azur, Nice, France

**Keywords:** membranous nephropathy, rituximab, autoimmunity, immunomonitoring, PLA2R1 autoantibodies, KDIGO (Kidney Disease: Improving Global Outcomes), immunosuppressive therapy, nephrotic syndrome

## Abstract

Primary membranous nephropathy (pMN) is an auto-immune disease characterized by auto-antibodies targeting podocyte antigens resulting in activation of complement and damage to the glomerular basement membrane. pMN is the most common cause of nephrotic syndrome in adults without diabetes. Despite a very heterogeneous course of the disease, the treatment of pMN has for many years been based on uniform management of all patients regardless of the severity of the disease. The identification of prognostic markers has radically changed the vision of pMN and allowed KDIGO guidelines to evolve in 2021 towards a more personalized management based on the assessment of the risk of progressive loss of kidney function. The recognition of pMN as an antibody-mediated autoimmune disease has rationalized the use immunosuppressive drugs such as rituximab. Rituximab is now a first line immunosuppressive therapy for patients with pMN with proven safety and efficacy achieving remission in 60-80% of patients. For the remaining 20-40% of patients, several mechanisms may explain rituximab resistance: (i) decreased rituximab bioavailability; (ii) immunization against rituximab; and (iii) chronic glomerular damage. The treatment of patients with rituximab-refractory pMN remains controversial and challenging. In this review, we provide an overview of recent advances in the management of pMN (according to the KDIGO 2021 guidelines), in the understanding of the pathophysiology of rituximab resistance, and in the management of rituximab-refractory pMN. We propose a treatment decision aid based on immunomonitoring to identify failures related to underdosing or immunization against rituximab to overcome treatment resistance.

## 1 Introduction

Primary membranous nephropathy (pMN) is an autoimmune disease affecting kidney glomerulus and the most common cause of nephrotic syndrome in non-diabetic Caucasian adults. The formation of subepithelial immune deposits and complement activation are responsible for the functional impairment of the podocyte, leading to the onset of the nephrotic syndrome (1, 2). The course of the disease is highly variable, ranging from spontaneous remission to persistent proteinuria or end-stage renal disease (3, 4). The treatment of pMN has long been based on a uniform management of patients with a 6-month supportive therapy period (5), which can lead to the persistence of a nephrotic syndrome that can be complicated in few cases by long-term renal failure (3, 4). The recent identification of prognostic markers drastically changed the vision of pMN and allowed KDIGO Clinical Practice Guideline for the Management of Glomerular Diseases to evolve in 2021 towards a more personalized management based on the assessment of the risk of progressive loss of kidney function (6).

The recognition of pMN as an autoantibody-driven disease and the identification of podocyte target antigens have been major advances in the diagnosis, prognosis and follow-up of patients with pMN. In addition to the M-type phospholipase A2 receptor type 1 (PLA2R1) and thrombospondin type-1 domain-containing 7A (THSD7A), other new antigens have recently been identified (7). These findings have rationalized the use of B-cell depleting agents such as rituximab, a chimeric monoclonal antibody targeting CD20. Over the last decade, rituximab became a first line therapy for pMN with proven safety and efficacy (8–10). However, 20 to 40% of patients do not respond to the first course of rituximab (8–10) and 5 to 28 % of patients relapse after a remission period (9–12). A more personalized approach is needed for these patients to understand the causes of treatment failure and to propose alternative treatment options. Recently described markers can predict the response to rituximab and new promising treatment options may help to overcome rituximab resistance. This review covers recent advances in the management of pMN and rituximab-refractory pMN, and proposes a therapeutic algorithm for an optimal and personalized management of pMN.

## 2 Management of Primary Membranous Nephropathy

### 2.1 New Advances in Treatment

The KDIGO 2012 Clinical Practice Guideline for the Management of Glomerular Diseases recommended a supportive therapy with inhibitors of the renin angiotensin aldosterone system for at least six months before initiating immunosuppressive therapy in nephrotic pMN without serious complications (5). This uniform management strategy has been a source of controversy given the highly variable evolution of the disease. The KDIGO 2021 Clinical Practice Guideline for the Management of Glomerular Diseases have been modified taking into account the numerous advances in the field of pMN since the previous issue in 2012 (6). Important progress has been made most notably in the identification of prognostic markers. Thus, the new guidelines propose to categorize patients with pMN and nephrotic syndrome according to their risk of kidney disease progression in order to provide personalized management. However, several additional markers can help to stratify disease severity as well as response to treatment.

### 2.2 Biomarkers of Disease Severity

**Figure 1:**
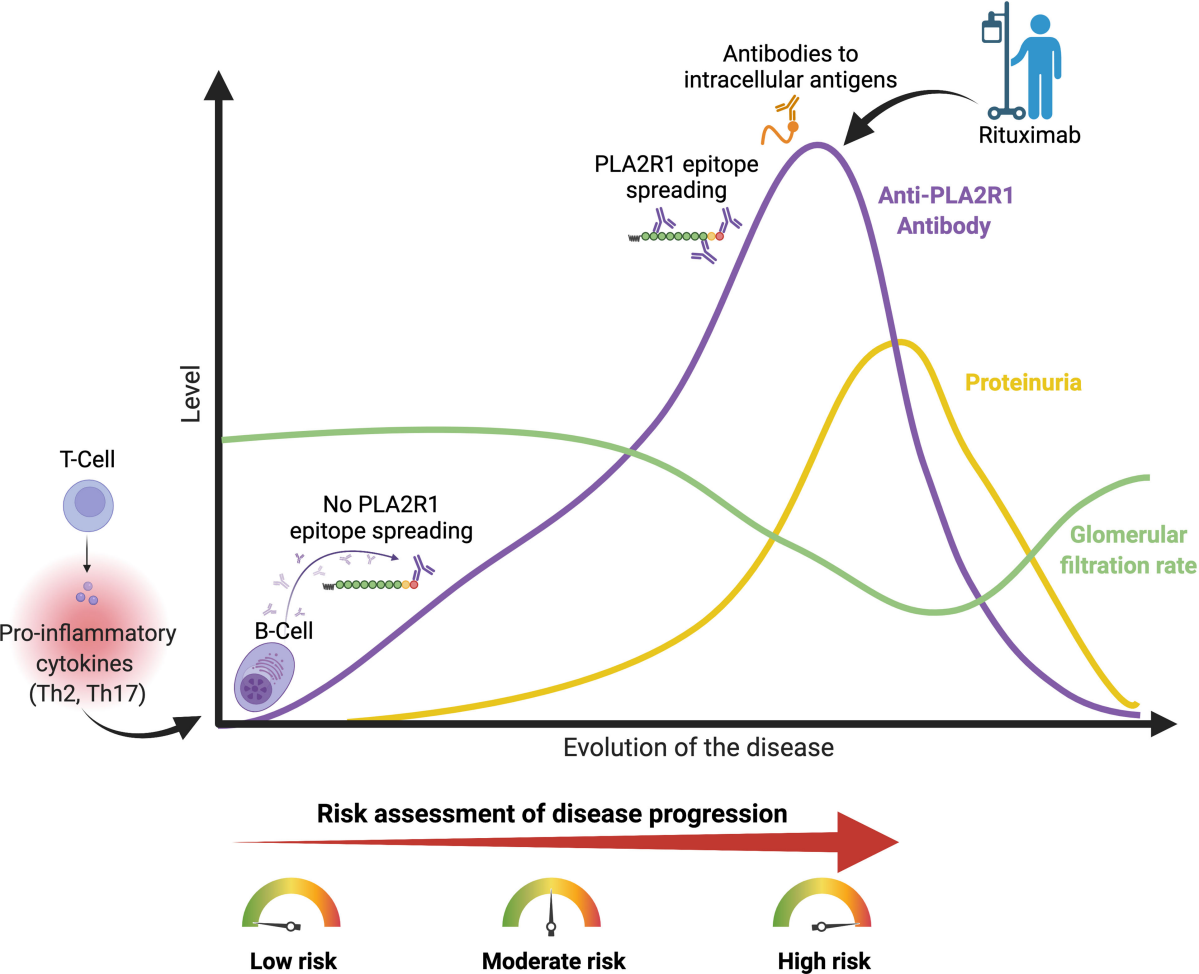
Risk of disease progression in membranous nephropathy. PLA2R1, phospholipase A2 receptor type 1. Th2 and Th17 pro-inflammatory cytokines are increased in patients with pMN (13, 14). High anti-PLA2R1 antibody titer, positive epitope spreading, antibodies to intracellular antigens, high proteinuria despite optimal supportive care and deterioration of kidney function are associated with the risk of disease progression. Urine low-molecular-weight proteins and urinary IgG excretion have been shown to correlate with disease progression in patients with pMN, but these markers are rarely used in daily practice. Figure created with BioRender.com.

The risk of persistent nephrotic syndrome or kidney disease progression should be considered as a combination of factors (**Figure 1**). A management algorithm based on these risk factors has been proposed in the KDIGO 2021 guidelines and in a recent review (6, 15).

#### 2.2.1 Proteinuria Level

The severity of the nephrotic syndrome and more specifically the level of proteinuria is associated with the risk of deterioration of renal function (4, 16). Patients with proteinuria >8 g/d for more than six months are at greater risk of impaired kidney function in the long term (16). Therefore, immunosuppressive therapy should be considered in patients with persistent high proteinuria despite a well conducted supportive therapy (6).

#### 2.2.2 Glomerular Filtration Rate

Reduced renal function defined as a glomerular filtration rate (GFR) < 60 ml/min/1.73m^2^ at diagnosis is associated with a higher risk of progression to renal failure (4, 16, 17). Patients with deteriorating kidney function may therefore immediately be treated with immunosuppressive therapy. In this case, rituximab and cyclophosphamide are recommended as first-line therapies in the KDIGO 2021 guidelines (6).

#### 2.2.3 Urine Low-Molecular-Weight Proteins and Urinary IgG Excretion

Urinary excretion of beta-2 microglobulin (uβ2m) and alpha-1 microglobulin (uα1m) – i.e. low-molecular-weight proteins – has been shown to correlate with disease progression and the evolution of renal function in patients with pMN (18–20). Urinary IgG excretion has been shown to predict the evolution of renal function in pMN patients (20, 21). However, these markers are rarely used in daily practice.

#### 2.2.4 Phospholipase A2 Receptor Type 1 Antibody Titer

In 2009, PLA2R1 was identified as the major podocyte antigen in 70-80% of pMN patients (22). Anti-PLA2R1 antibodies are highly specific for the diagnosis of pMN (23). Thus, according to the KDIGO 2021 guidelines, a kidney biopsy is not required to confirm the diagnosis of pMN in patients with nephrotic syndrome and a positive anti-PLA2R1 antibody test (6). PLA2R1 antibody titers correlate with disease activity, as antibodies usually disappear during spontaneous or treatment-induced remission and reappear in relapse (24).

Anti-PLA2R1 antibody titers also correlate with disease prognosis: a high titer at diagnosis was associated with a lower rate of spontaneous (25–29) or treatment-induced clinical remission (30). Patients with high anti-PLA2R1 titer achieved clinical remission significantly later than patients with low titer (26). In addition, a high anti-PLA2R1 titer at diagnosis was associated with a higher risk of long-term renal impairment (31, 32) and a higher risk of developing nephrotic syndrome in previously non-nephrotic patients (33). However, the thresholds used to define a high titer varied in these studies, it therefore remains unclear what specific antibody level should be used to predict the risk of disease progression. An anti-PLA2R1 antibody titer greater than 50 or 150 RU/mL appears to be reasonable to define a high antibody titer (6, 15). Between 50 and 150 RU/mL, the PLA2R1 epitope spreading analysis may be an important additional tool to assess patient prognosis. On the contrary, a low titer or the absence of anti-PLA2R1 antibody in patient with PLA2R1-associated pMN is correlated with a higher likelihood of spontaneous clinical remission (25–29, 33, 34). The decrease or disappearance of autoantibodies during follow-up usually precedes clinical remission (24, 30). Therefore, low baseline and decreasing anti-PLA2R1 antibody titers during follow-up strongly predict spontaneous remission, thus favoring supportive therapy alone. High baseline or increasing anti-PLA2R1 antibody titers, on the other hand, are associated with persistent nephrotic syndrome and progressive loss of kidney function, which should lead to the prompt initiation of immunosuppressive therapy (6).

Since re-emergence or increase in antibody titers precedes a clinical relapse, anti-PLA2R1 antibody titers should also be followed after immunosuppressive therapy (24, 30). The immunological response to immunosuppressive therapy may guide the adaptation of the treatment regimen. The KDIGO 2021 guidelines recommend monitoring of anti-PLA2R1 antibody titers with a first assessment three months after the start of therapy. In the case of stability or increased antibody titer, additional doses of treatment might be proposed (6).

#### 2.2.5 Phospholipase A2 Receptor Type 1 Antibody Epitope Spreading

Epitope spreading refers to the development of an immune response directed against epitopes distinct from the dominant epitope, without any cross-reactivity. This process is common in the fight against infectious agents. The epitopes may be located on the same antigen (called intramolecular epitope spreading) or on a different antigen (called intermolecular epitope spreading) (35).

This phenomenon was described in Heymann nephritis, a rat model of membranous nephropathy (36). In this model, rats immunized solely with the dominant megalin epitope gradually developed immunization against other epitopes of megalin, which was associated with a worsening of the disease. The same process may happen in humans with PLA2R1. PLA2R1 consists of several domains: (i) a cysteine-rich domain (CysR), (ii) a fibronectin type II domain (FnII) and (iii) eight C-type lectin domains (CTLD1 to CTLD8) (37). We and others have identified several different epitopes that can be recognized by anti-PLA2R1 antibodies (37–40): CysR (the dominant epitope), CTLD1, CTLD7 and CTLD8. In one of the studies, 67% of patients with pMN associated with anti-PLA2R1 antibodies had antibodies to CTLD1 and/or CTLD7 in addition to the dominant epitope CysR, defining intramolecular epitope spreading. Patients with epitope spreading had higher proteinuria level at diagnosis, poorer renal survival and a lower rate of spontaneous remission rate compared to patients without epitope spreading (37). In the GEMRITUX cohort, non-spreader patients had a spontaneous remission rate of 45% at six months while spreaders had a rate of 5% (41). Epitope spreading was an independent risk factor for poor renal prognosis (defined as persistence of proteinuria > 4 g/g and/or a GFR estimated by the CKD-EPI formula < 45 ml/min/1.73m^2^ at the end of follow-up) and treatment failure (37, 41).

However, a recent study using a different detection technique demonstrated no additional predictive value of epitope spreading (40). Probably due to this lack of consensus, the epitope spreading did not enter in the risk-based treatment algorithm of pMN proposed by the KDIGO 2021 guidelines. More studies are needed to clearly establish the predictive value of epitope spreading for patients with PLA2R1-associated pMN (42).

#### 2.2.6 Anti-Thrombospondin Type-1 Domain-Containing 7A Antibody Titer

Anti-THSD7A antibodies are present in approximately 3% of pMN patients (43). Similarly to anti-PLA2R1 antibodies, anti-THSD7A antibody titers correlate with disease activity (44, 45). However, due to the rarity of THSD7A-associated pMN, data on the prognostic value of anti-THSD7A antibody titers are limited (44). Anti-THSD7A antibody monitoring is not included in the management algorithm proposed by the KDIGO 2021 guidelines, but antibody titer-based management should probably also be applied to patients with THSD7A-associated pMN.

The mechanism of epitope spreading has also been described in THSD7A-related pMN but due to lack of statistical power, the impact of spreading on prognosis could not be evaluated (46).

#### 2.2.7 Cytokine Profile and Environment

Environmental factors may play a role in the pathophysiology of pMN. In China, the incidence of pMN was correlated to the level of exposure to fine particles in the air (47, 48). Based on a functional approach, we have shown that: (i) pMN patients had higher levels of pro-inflammatory Th2 and Th17 cytokines than healthy subjects, and (ii) that patients with high levels of Th17 cytokines lived in urbanized areas highly exposed to fine particles in the air (13). Increased levels of Th17 cytokines were associated with more venous thromboembolic events and a 10.5-fold higher risk of relapse (13). However, the cytokine profile is not measured in daily clinical practice and this criterion is not included in the management algorithm proposed by the KDIGO 2021 guidelines (6).

#### 2.2.8 Antibodies to Intracellular Antigens

Antibodies targeting different intracellular podocyte antigens such as aldose reductase, superoxide dismutase 2, and α-enolase have been identified in a significant number of patients with pMN (49, 50). In PLA2R1-associated pMN, the immunization against superoxide dismutase 2 and α-enolase was associated with a lower rate of clinical remission and a greater impairment of renal function (51). Patients with PLA2R1 epitope spreading were at higher risk of immunization against these intracellular antigens (51). Immunization against intracellular antigens could be the result of a multi-hit pathogenic mechanism. First, antibodies recognize a dominant epitope on a primary autoantigen (e.g. CysR on PLA2R1). Subsequently (i) intramolecular epitope spreading within the primary autoantigen may occur, and (ii) intermolecular epitope spreading with the formation of antibodies could target intracellular autoantigens (e.g. aldose reductase, anti-superoxide dismutase 2 and anti-α-enolase). These intra- and intermolecular epitope spreading would lead to an amplification of the immune response against the podocyte and, potentially, a more severe disease progression (51). However, testing for the antibodies against intracellular antigens is not performed in daily clinical practice and this criterion is not included in the management algorithm proposed by the KDIGO 2021 guidelines (6).

### 2.3 Rituximab in Primary Membranous Nephropathy

To date, the alkylating agents (e.g. cyclophosphamide) are the only treatment with proven efficacy to prevent end stage kidney disease (ESKD) and death (52–54). The basis for recommendations on these treatments comes from studies conducted three decades ago in which alkylating agents improved nephrotic syndrome and renal disease progression over non-immunosuppressive antiproteinuric therapy (52, 53). However, alkylating agents combined with corticosteroids are associated with a risk of serious infections, late malignancy, infertility and other severe adverse events (55). Rituximab appears to be safer, and allows a high rate of clinical remission, which has been associated with preservation of renal function on the long term.

Rituximab is an anti-CD20 chimeric monoclonal antibody, which can trigger B-cell death by apoptosis, complement-mediated cytotoxicity, and antibody-dependent cellular cytotoxicity (10).

Since the first report of patients with pMN treated with rituximab in 2002 (56), rituximab has progressively emerged as the first choice treatment for pMN due to its high safety and efficacy. In several non-randomized studies, the remission rate for pMN patients treated with rituximab ranged from 57% to 89% (11, 12, 56, 57).

In a large retrospective observational cohort study, van den Brand et al. analyzed outcomes of 100 patients treated with rituximab compared with 103 patients treated with glucocorticoid plus oral cyclophosphamide. Over a median follow-up of 40 months, the rituximab group had significantly fewer adverse events than the cyclophosphamide/glucocorticoid group. Although the cumulative incidence of partial remission was lower in the rituximab group, rates of complete remission and a composite end point of doubling of serum creatinine, end-stage kidney disease, or death did not differ significantly between groups (55).

In the multicentric randomized controlled trial GEMRITUX, rituximab (2 infusions of 375 mg/m^2^ on day 1 and 8) combined with a non-immunosuppressive antiproteinuric treatment (NIAT) was compared with NIAT alone (8). At six months, there was no significant difference between the groups in the rate of clinical remission. However, in the extended follow-up (after a median follow-up of 17 months), a significant difference was reported, with clinical remission occurring in 65% of the NIAT-rituximab group but only in 34% of the NIAT alone group (*p*<0.01). In addition, anti-PLA2R1 antibodies depletion rates were greater in the NIAT-rituximab group than in the NIAT alone group at month-3 (56% and 4%, respectively, *p*<0.001) and at month-6 (50% and 12%, respectively, *p*=0.004). The delayed efficacy of rituximab in the GEMRITUX study could therefore be explained by the fact that immunological remission precedes clinical remission by several months (30).

In the multicentric randomized controlled trial MENTOR, rituximab (2 infusions of 1 g on day 1 and 15) was compared to cyclosporine. Rituximab was as effective as cyclosporine in inducing clinical remission at 12 months, but discontinuation of cyclosporine after 12 months resulted in an increased rate of relapse, resulting in a higher clinical remission rate at 24 months in the rituximab group than in the cyclosporine group (60% in the rituximab group *vs* 20% in the cyclosporine group, *p*<0.001). The decrease in autoantibody titer was faster, greater, and more durable in the rituximab group than in the cyclosporine group. Serious adverse events occurred in 11 patients (17%) in the rituximab group and in 20 (31%) in the cyclosporine group (9).

More recently, a randomized controlled trial RI-CYCLO compared the efficacy of cyclic cyclophosphamide/glucocorticoid regimen to rituximab (two infusions of 1 g on day 1 and 15) in inducing clinical remission (58). At 12 months, 23/37 (62%) patients in the rituximab group and 27/37 (73%) in cyclophosphamide/glucocorticoid group had a complete or partial remission. At 24 months, probabilities of complete and partial remission were comparable between the two groups. The frequency of serious adverse events was comparable between the groups (19% of patients in the rituximab group and in 14% of patients in cyclophosphamide/glucocorticoid group). The authors concluded that trial found no evidence of more benefit or less harm associated with rituximab compared with a cyclophosphamide/glucocorticoid regimen in the treatment of pMN.

All these studies show remarkable efficacy of rituximab with limited side effects compared to cyclophosphamide or cyclosporine. This supports the use of rituximab as a first-line treatment for pMN.

### 2.4 Calcineurin Inhibitors in Primary Membranous Nephropathy

Calcineurin inhibitors (CNIs) (e.g. cyclosporine and tacrolimus) are immunosuppressive therapies that inhibit T-cell activation. CNIs have also been shown to have an immunomodulatory effect by inhibiting the Th17 immune response, which is implicated in the development of autoimmune diseases such as pMN (59–61). CNIs have been used for many years in the management of pMN.

In the MENTOR trial, cyclosporine was less effective than rituximab in achieving long-term persistent clinical remission (9). Furthermore, cyclosporine was associated with a high incidence of relapse after discontinuation of treatment (53% in the cyclosporine group *vs* 5% in the rituximab group) and frequent side effects.

Ramachandran et al. compared the long-term efficacy of a combination of cyclophosphamide and glucocorticoid for six months to a combination of tacrolimus for one year and prednisone for six months. At the end-point of six years after the beginning of treatment, 62% of participants in the cyclophosphamide/glucocorticoid group and 28% in the tacrolimus group maintained remission without relapse, and 88% of patients in the cyclophosphamide/glucocorticoid group *vs* 53% of patients in the tacrolimus group were in remission. This study confirmed the long-term superiority of six months of cyclophosphamide/glucocorticoid therapy *vs* one year of tacrolimus (62).

In summary, these studies show that CNI monotherapy regimens are less likely to achieve long-term clinical remission than rituximab or cyclophosphamide/glucocorticoid-based protocols. For this reason, the KDIGO 2021 guidelines suggest that CNI monotherapy is justifiable only in patients with a normal glomerular filtration rate and a moderate risk of disease progression – as these patients have less severe disease with a greater likelihood of achieving remission – in order to shorten the period of proteinuria (6).

## 3 Rituximab-Refractory Membranous Nephropathy

**Figure 2:**
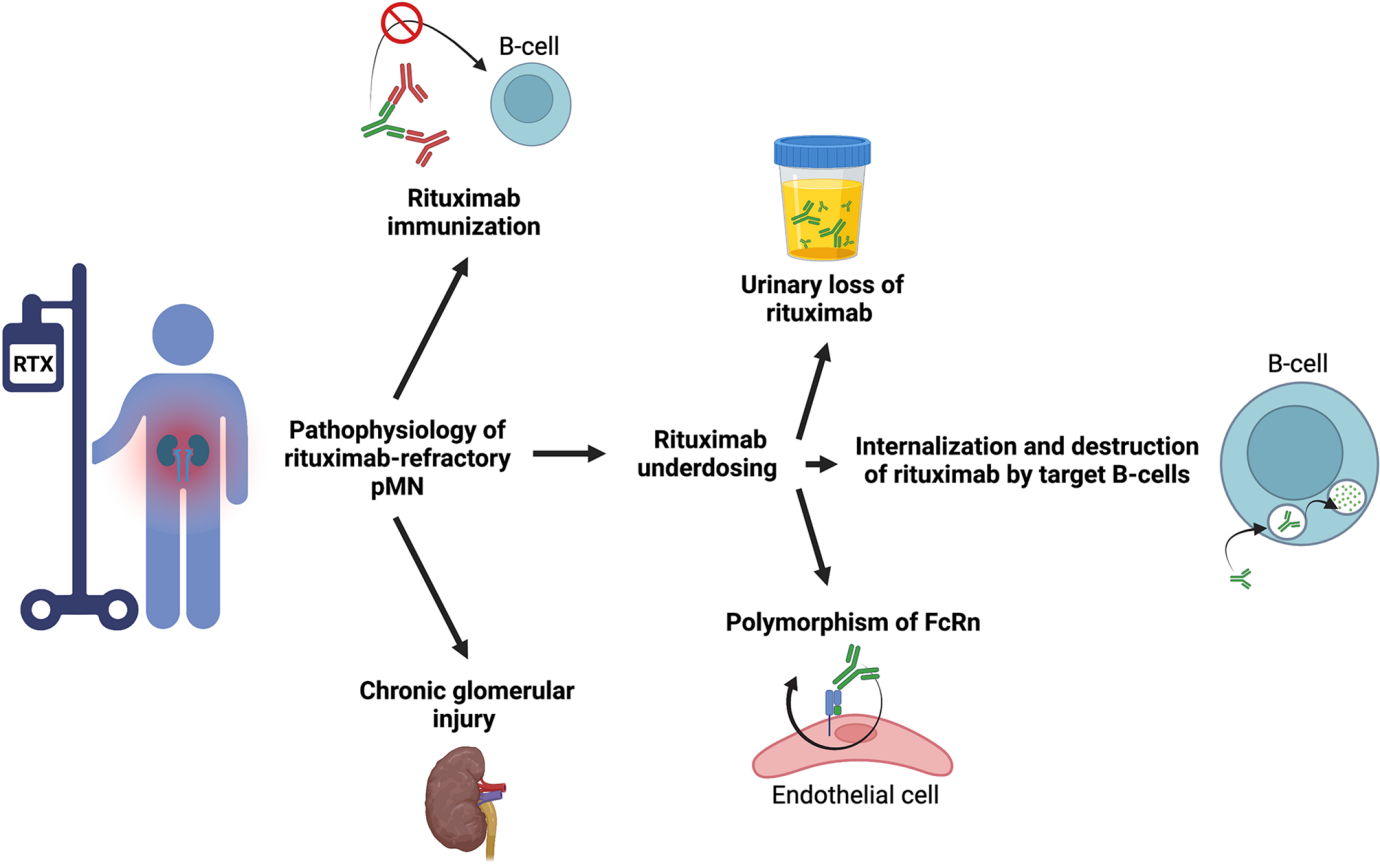
Main causes of rituximab resistance in membranous nephropathy. ADAs, anti-drug antibodies; FcRN, neonatal Fc receptor for IgG; PLA2R1, phospholipase A2 receptor type 1; RTX, rituximab. Figure created with BioRender.com.

Rituximab-refractory pMN may be defined by the absence of clinical and/or immunological remission (i.e. antibody titer below the detection threshold by ELISA or a negative indirect immunofluorescence assay) after a rituximab course. Understanding the mechanisms behind treatment failure allows the identification of additional therapeutic strategies for rituximab-refractory pMN. The main mechanisms behind treatment failure are summarized in **Figure 2**.

### 3.1 Causes of Rituximab Resistance

#### 3.1.1 Rituximab Underdosing

Rituximab underdosing is not uncommon in pMN (63, 64). Indeed, rituximab bioavailability is significantly decreased in pMN compared to other non-nephrotic autoimmune diseases due to the binding of rituximab to albumin and its wasting in the urine (63, 65, 66). We have shown that rituximab was undetectable three months (month-3) after rituximab infusion (two 1 g infusions two weeks apart) in 56% of nephrotic pMN, these patients were less likely to achieve clinical and immunological remission (64). Patients with the most severe nephrotic syndrome – with a baseline albumin level of less than 22.5 g/L – were more likely to have an undetectable serum rituximab level at month-3 (64). Serum rituximab level can be measured by ELISA (which costs about 40€ in France). However, this technique is not routinely available in all centers. If required, it is recommended to refer to an expert center. In addition to urinary excretion of rituximab, internalization and destruction of rituximab by target B-cells and polymorphism of the neonatal Fc receptor for IgG (FcRn) – which protects antibodies from degradation by lysosomes and thus reduces their clearance by allowing their recycling in the cellular environment – may also decrease rituximab bioavailability (67–70).

#### 3.1.2 Rituximab Immunization

Rituximab is a chimeric monoclonal antibody including human IgG1 constant regions and a murine anti-human CD20 variable region. The use chimeric monoclonal antibodies may be complicated by the development of anti-drug antibodies (ADAs) such as anti-rituximab antibodies. Twenty-three to 43% of patients treated with rituximab develop anti-rituximab antibodies during follow-up (57, 71). These ADAs neutralized rituximab activity (complement-dependent cytotoxicity and antibody-dependent cell-mediated cytotoxicity) in 8 of 10 patients (80%). Anti-rituximab antibodies resulted in faster B-cell reconstitution and a higher rate of relapse (71). Therefore, in patients previously treated with rituximab, anti-rituximab antibodies should be systematically tested before starting a new course of rituximab. Anti-rituximab antibodies can be measured by ELISA (which costs about 40€ in France). However, this technique is not routinely available in all centers. If required, it is recommended to refer to an expert center.

#### 3.1.3 Chronic and Irreversible Damage to the Glomerular Filtration Barrier

The presence of fibrous glomerular damage may be responsible for significant proteinuria. In patients who are refractory to multiple immunosuppressive therapies, it may be difficult to distinguish between primary immunosuppressive resistance and resistance secondary to chronic and irreversible glomerular damage. In these patients, a repeat kidney biopsy and monitoring of immunological activity (anti-PLA2R1 or anti-THSD7A titers) may be useful to distinguish between patients with an immunologically active disease who may benefit from additional immunosuppressive therapy (24, 44, 45), and patients with extensive chronic histologic lesions in whom additional immunosuppressive therapy is futile.

### 3.2 Management of Rituximab-Refractory Membranous Nephropathy

The KDIGO 2021 guidelines suggest that patients who are refractory to a first course of rituximab should receive a second course of rituximab and calcineurin inhibitors (CNIs) or cyclophosphamide and glucocorticoids (6). However, this does not take into account the cause of rituximab resistance. An approach based on immunomonitoring of rituximab and anti-rituximab antibodies would allow a more targeted management.

#### 3.2.1 Optimized Supportive Therapy

In order to reduce proteinuria and urinary loss of rituximab, the supportive therapy should be optimized. It should include a renin-angiotensin-aldosterone system inhibitor (angiotensin-converting enzyme inhibitor or angiotensin II receptor blocker), a diuretic as well as a low-sodium diet (< 2 g/d) (6, 72, 73). The control of extracellular overload and arterial blood pressure are major components of the management of the nephrotic syndrome; systolic blood pressure should be < 120 mmHg (6). In the absence of renal failure, protein intake should be 0.8 to 1 g/kg/d (if proteinuria > 5 g/d: add 1g per 1g of protein loss) (6). High- or low-protein diets are not recommended. A low-fat diet is recommended for patients with high cholesterol to prevent cardiovascular complications (6).

#### 3.2.2 Rituximab Dosing

In nephrotic patients, the bioavailability of rituximab is decreased due to the elimination of rituximab in the urine (63, 64). Uncertainty remains regarding the rituximab dosing protocol to be used in nephrotic patients.

In the GEMRITUX study, B-cells were not fully depleted six months after rituximab treatment, suggesting that the dose used (two 375 mg/m^2^ injections one week apart) was suboptimal (8). This lack of B-cell depletion may explain the lack of significant difference in the 6-month clinical remission rate between the rituximab-NIAT group and the NIAT alone group (8).

More recently, we have shown that a high-dose rituximab regimen (two 1000 mg injections two weeks apart) was more effective on B-cell depletion and was associated with a higher clinical remission rate than the GEMRITUX regimen (10). The median residual rituximab level at month-3 was higher with the high-dose rituximab regimen compared to the GEMRITUX regimen and was correlated with higher rates of clinical remission.

Two recent randomized controlled trials also used a high-dose rituximab regimen (i.e. two 1000 mg injections). Remission rates at 6-month were comparable with high-dose rituximab in MENTOR and low-dose regimen in GEMRITUX despite higher baseline anti-PLA2R1 titers in MENTOR (8, 9). Remission rates at 6-month were higher with high-dose rituximab in RI-CYCLO compared to low-dose regimen in GEMRITUX despite comparable baseline anti-PLA2R1 titers (8, 58). These data would also favor high-dose rituximab.

Since patients with severe nephrotic syndrome are at higher risk of rituximab underdosing and treatment failure (64), higher initial doses of rituximab in these patients may limit the risk of treatment resistance and increase the likelihood of remission by fully depleting B-cells. High-dose rituximab regimen also appears to be required in patients with epitope spreading, while a low-dose of rituximab regimen may be sufficient for patients with anti-PLA2R1 activity limited to CysR (10). Indeed, all patients with antibodies restricted to the CysR domain (non-spreaders) entered into remission at last observation carried forward regardless of the rituximab protocol administered, while patients with epitope spreading had a higher likelihood of remission with a high-dose rituximab regimen (10). Similarly, patients with high anti-PLA2R1 antibody titers at baseline achieved clinical remission more frequently with the high-dose rituximab regimen (10). A clinical trial is underway in patients with PLA2R1-associated pMN to compare the efficacy of the GEMRITUX regimen (low-dose rituximab after six months of anti-proteinuric therapy) to a personalized management (stratifying the patients according to their epitope spreading status at month-0 and month-6 and treating them accordingly with either low- or high-dose rituximab regimen) (42).

#### 3.2.3 Repeated Rituximab Injections

Reinfusion of rituximab can induce immunological and clinical remission in patients considered refractory to rituximab (74, 75). Dahan et al. described ten patients who were refractory to an initial course of rituximab and who were retreated with rituximab resulting in remission in eight of them (74). We have further expanded this study by showing that patients who failed to respond to a first course of rituximab and who did not develop anti-rituximab antibodies responded to repeated courses of rituximab while those who developed antibodies only responded to treatment with human or humanized anti-CD20 monoclonal antibodies (75).

For patients with PLA2R1-associated pMN, immunomonitoring of anti-PLA2R1 antibodies may be beneficial for guiding rituximab therapy. In the absence of immunological remission, repeated rituximab injections at three and/or six months after the start of treatment increased clinical remission rate at 12 months to 91% (76). KDIGO 2021 guidelines recommend anti-PLA2R1 antibody monitoring at month-3 and -6 and administering an additional rituximab dose to patients with persistent or increasing anti-PLA2R1 titers (6).

Since serum rituximab levels are lower during follow-up in patients with nephrotic pMN and since undetectable rituximab levels at month-3 are associated with a risk of treatment failure, rituximab immunomonitoring may be useful in treatment decision-making (63, 64). Patients with undetectable serum rituximab levels (< 2 µg/mL) at month-3 did not achieve clinical remission at month-6 if proteinuria was greater than 5.5 g/d at month-3 (64). In these patients, additional early doses of rituximab must be considered. In order to limit the urinary loss of rituximab in patients with active nephrotic syndrome, it is also important to ensure that supportive therapy is provided at the maximum tolerated dose (no extracellular overload without serum creatinine increase >30% and orthostatic hypotension).

#### 3.2.4 Human and Humanized Anti-CD20 Antibodies

New generations of humanized or fully human anti-CD20 antibodies have been developed. Obinutuzumab and ofatumumab are directed to a different epitope on CD20 and have higher affinity for CD20 than rituximab (77, 78). Obinutuzumab is a humanized and glycoengineered type II anti-CD20 monoclonal antibody. The glycan tree of the Fc fragment of obinutuzumab was modified to enhance antibody-dependent cellular cytotoxicity (78). Ofatumumab is a human type I anti-CD20 antibody that activates complement-dependent cytotoxicity more effectively than rituximab (77). Obinutuzumab and ofatumumab have superior *in vitro* and *in vivo* B-cell cytotoxicity and lower risk of immunization compared to rituximab (69, 77). These new monoclonal antibodies have shown efficacy in some autoimmune or inflammatory diseases (such as rheumatoid arthritis, systemic autoimmune diseases and chronic inflammatory demyelinating polyneuropathy) after the development of resistance to chimeric monoclonal antibodies following the appearance of ADA (79, 80). Anti-rituximab antibodies cross-reacted with obinutuzumab or ofatumumab in only 20% of patients with ADA (71). In a series of four cases with rituximab-refractory pMN, obinutuzumab and ofatumumab were effective in achieving clinical and immunological remission (75, 81–83). However, randomized controlled trials comparing ofatumumab or obinutuzumab to rituximab in patients with non-Hodgkin lymphoma have not found superiority of these new monoclonal antibodies over rituximab (84, 85). Randomized and well-powered clinical trials are needed to evaluate the efficacy and the optimal dose of humanized or human anti-CD20 in patients with pMN who develop anti-rituximab antibodies.

#### 3.2.5 Calcineurin Inhibitors in Combination With Rituximab

The STARMEN trial, compared the effectiveness of a 6-month induction course with tacrolimus (followed by tapering over another three months) in combination with a single dose of rituximab (1 g) at six months with a 6-month cyclical therapy of methylprednisolone and cyclophosphamide (86). The tacrolimus/rituximab protocol was less effective than the cyclophosphamide/glucocorticoid protocol in achieving clinical remission at 24 months (58% *vs* 84%, respectively), while serious adverse events were similar in both groups. The immunological response (depletion of anti-PLA2R1 antibodies) was also significantly higher at three and six months in the cyclophosphamide/glucocorticoid group (77% and 92%, respectively) than in the tacrolimus/rituximab group (45% and 70%, respectively). It is important to note that the addition of rituximab was delayed by six months in this study, which may explain the limited effectiveness of this combination. Moreover, this study has some limitations, including a higher proportion of male patients and higher anti-PLA2R1 antibody titers in the tacrolimus/rituximab group than in the cyclophosphamide/glucocorticoid group.

A pilot trial combining rituximab (two infusions of 1 g on day 1 and 15, repeated at six months) and cyclosporine showed substantially higher rates of complete clinical remission and immunological response at 24 months than those observed with rituximab or cyclosporine alone in the MENTOR trial (87, 88).

Therefore, the combination of CNI/rituximab in patients refractory to rituximab because of underdosing may be of interest. In addition to their immunosuppressive and immunomodulating effects, CNIs may decrease the urinary loss of rituximab by causing glomerular arteriolar vasoconstriction. It is important to note that this combination is likely to result in significant immunosuppression with an increased risk of infection.

#### 3.2.6 Other Treatments

Plasma exchange, immunoabsorption, mycophenolate mofetil (MMF), adrenocorticotropic hormone (ACTH) and bortezomib have been proposed for the management of refractory pMN.

Plasma exchange and immunoabsorption have been proposed in the management of pMN to rapidly decrease the titers of autoantibodies. However, only small series have been reported in patients with severe or refractory pMN (83, 89, 90). Plasma exchange and immunoabsorption do not directly modulate B-cell proliferation and activity, so the patients additionally received immunosuppressive treatment. Plasma exchange and immunoabsorption combined with immunosuppressive therapy seem to allow a faster remission than immunosuppressive therapy alone in patients with severe disease or refractory to conventional treatment.

MMF has also been proposed for the management of refractory pMN. In a study of 16 patients with pMN, including 15 steroid-resistant, six alkylating agent-resistant, and five cyclosporine-resistant patients, treatment with MMF for a mean duration of eight months resulted in a halving of proteinuria in six patients and partial clinical remission in two patients (91).

ACTH was administered to five pMN patients resistant to initial treatment. Three patients had previously received CNIs, two cyclophosphamide, two steroids, four MMF, and one patient previously received rituximab. Two of the five patients achieved partial clinical remission on ACTH therapy and three patients achieved immunological remission (92).

Finally, several case studies have reported the efficacy of bortezomib – an anti-plasma cell agent – in combination with glucocorticoids in the treatment of pMN refractory to first-line immunosuppressants (93–95).

High quality data demonstrating the effectiveness of these treatments are lacking. Well-conducted randomized clinical trials are needed to assess the effectiveness of these treatments in the management of refractory pMN. Therefore, these treatments should not be used as first line therapy and their use should be systematically discussed with an expert center.

#### 3.2.7 Personalized Management

**Figure 3:**
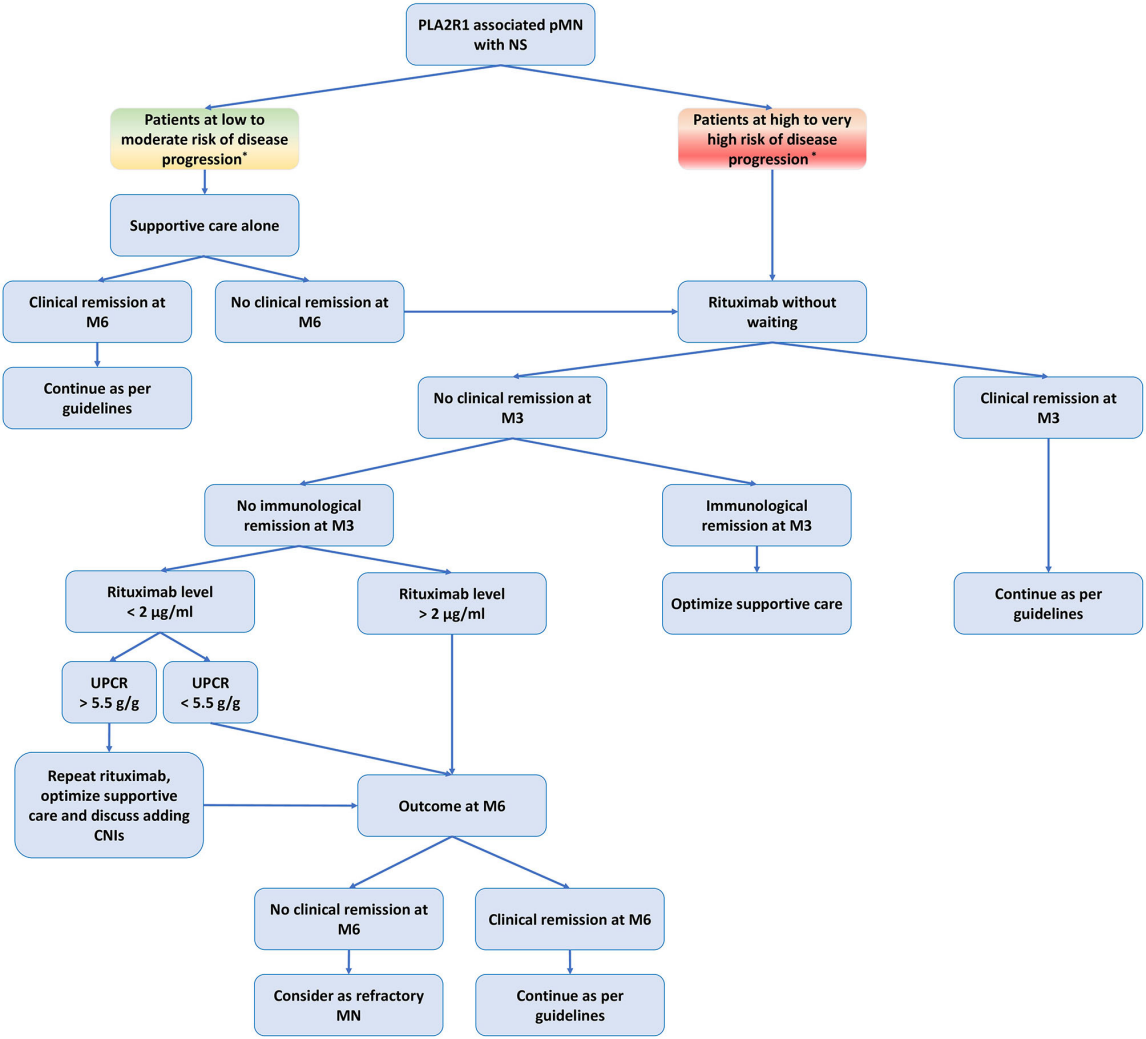
Treatment of primary membranous nephropathy based on risk assessment of disease progression and rituximab immunomonitoring. CNIs, calcineurin inhibitors; M3, month-3 (3 months after rituximab infusion); M6, month-6 (six months after rituximab infusion); NS, nephrotic syndrome; pMN, primary membranous nephropathy; PLA2R1, phospholipase A2 receptor type 1; RTX, rituximab; UPCR, urine protein/creatinine ratio. *Low to moderate risk of disease progression: negative epitope spreading and anti-PLA2R1 titer < 150 RU/ml, and no severe complication related to NS, and reduction of proteinuria under optimal supportive care and no deterioration of kidney function. High to very high risk of disease progression: positive epitope spreading, and/or anti-PLA2R1 titer > 150 RU/ml, and/or severe complication related to NS, and/or high proteinuria (> 8 g/d) despite optimal supportive care and/or deterioration of kidney function. Urine low-molecular-weight proteins and urinary IgG excretion have been shown to correlate with disease progression in patients with pMN, but these markers are rarely used in daily practice. Serum rituximab level analyzed by ELISA (LISA-TRACKER Duo Rituximab, Theradiag ^©^ Croissy Beaubourg, France) three months after the last injection. The limit of detection defined by the manufacturer is 2 µg/ml.

**Figure 4:**
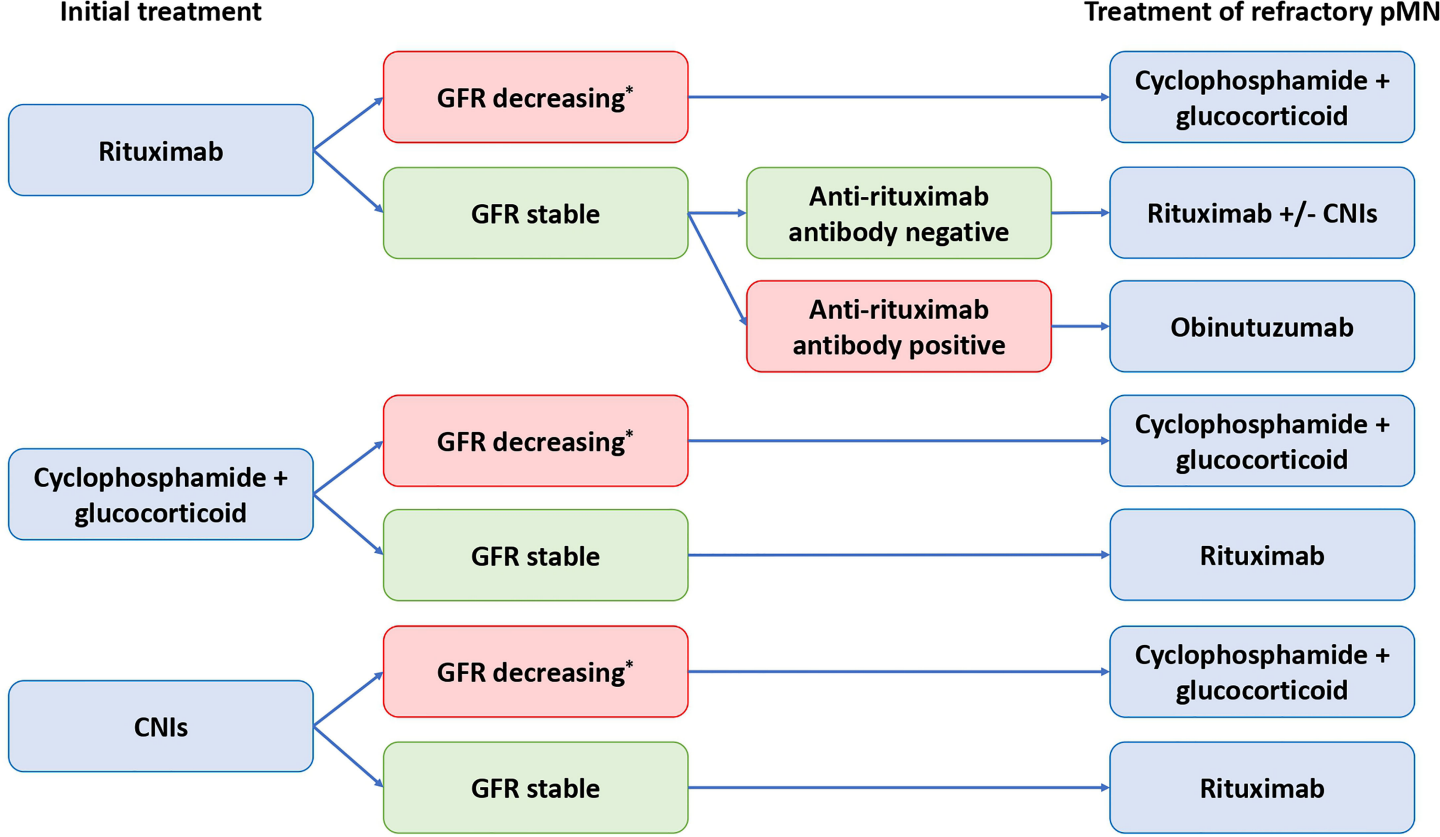
Algorithm for management of patients with treatment-resistant primary membranous nephropathy. CNIs, calcineurin inhibitors; GFR, glomerular filtration rate; pMN, primary membranous nephropathy. *directly related to membranous nephropathy. To date, the alkylating agents (e.g. cyclophosphamide) are the only treatment with proven efficacy to prevent end stage kidney disease (ESKD) and death.

**Figure 5:**
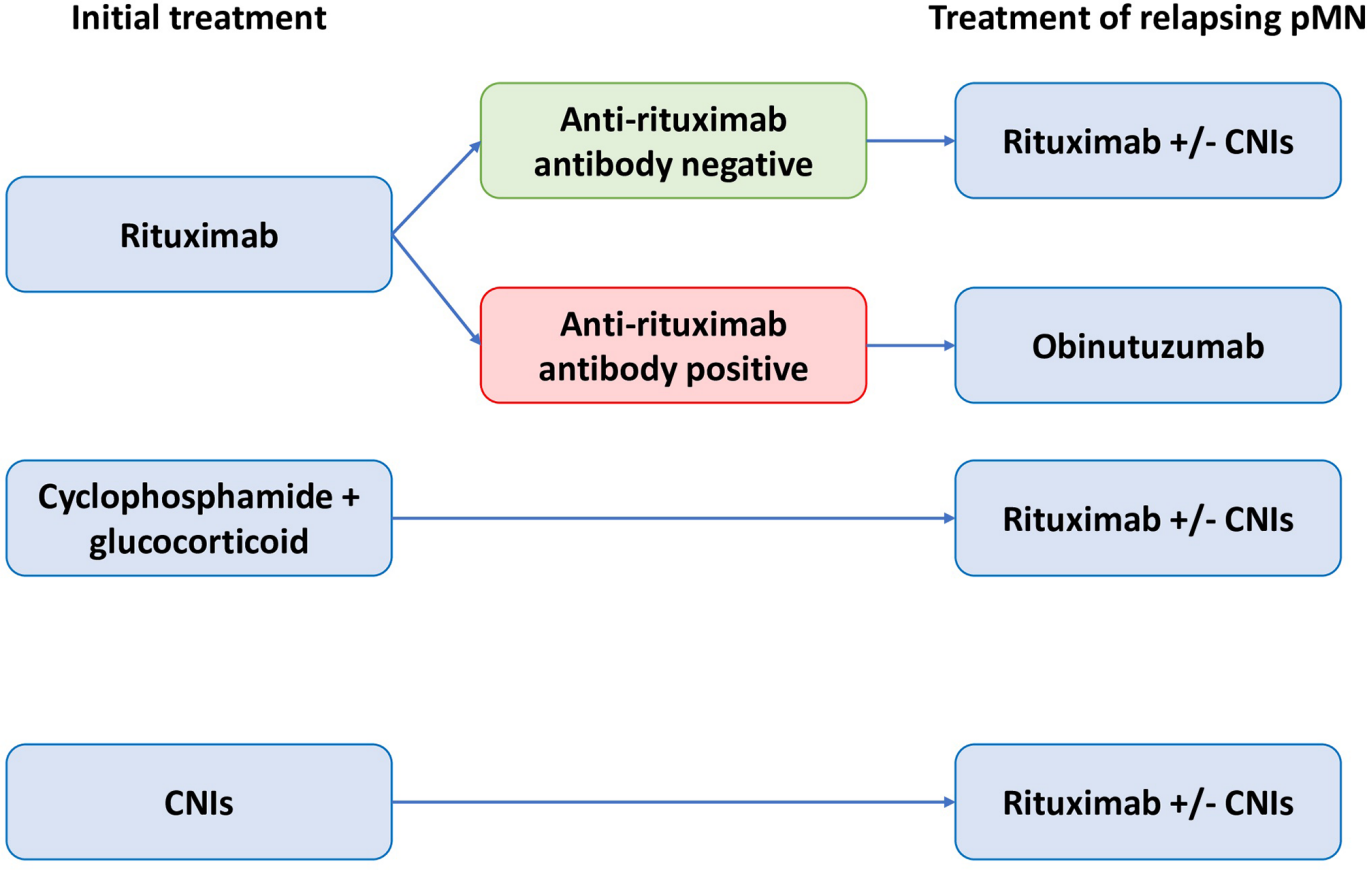
Algorithm for management of patients with relapsing primary membranous nephropathy. CNIs, calcineurin inhibitors; pMN, primary membranous nephropathy.

We propose a personalized management of pMN that takes into account (**Figures 3**–**5**): (i) the risk assessment of disease progression (see 2.2 Biomarkers of Disease Severity); (ii) a better understanding of the pathophysiology of rituximab-refractory pMN (see 3.1 Causes of Rituximab Resistance); and (iii) rituximab and ADAs immunomonitoring (see 3.2 Management of Rituximab-Refractory Membranous Nephropathy).

In patients with high or very high risk of disease progression, immunosuppressive therapy should be initiated promptly, whereas it may be delayed by 3 to 6 months in patients with low or moderate risk.

Immunomonitoring allows the personalization of the initial therapeutic management as well as the management of refractory or relapsing pMN with a fairly low cost (**Figures 3**–**5**).

We propose to assess the residual serum rituximab level three months after rituximab infusion to identify underdosed patients. In these patients, additional doses of rituximab should be considered as early as month-3 if proteinuria is greater than 5.5 g/d (64). Optimized supportive therapy is required to prevent urinary loss of rituximab. The addition of CNI may also be discussed.

We propose to measure anti-rituximab antibodies in rituximab-refractory pMN or relapsing pMN after rituximab in order to identify patients who should receive a treatment with human or humanized anti-CD20 monoclonal antibodies (**Figures 4**, **5**).

## 4 Perspectives in the Management of Membranous Nephropathy

### 4.1 Complement Inhibitors

The pathogenicity of complement in the development of pMN is well demonstrated (1, 2, 96). The complement is involved in the physiopathology of pMN, notably *via* the formation of the membrane attack complex C5b-9 following the activation of the classical pathway, the lectin pathway or the alternative pathway. C5b-9 forms a transmembrane pore that can cause osmotic lysis of the podocyte. C5b-9 can also cause damage to the glomerular filtration membrane by various other mechanisms, such as: (i) stimulation of the production of reactive oxygen species, proteases and prostanoids; (ii) modifications of the actin cytoskeleton and alterations of podocyte slit diaphragm; (iii) stimulation of the production of TGF-β and extracellular matrix components and (iv) limitation of podocyte cell proliferation (97). Blocking complement activation and C5b-9 formation is therefore an attractive therapeutic option. As IgG4 can activate the lectin pathway (98), the use of treatments that inhibit the common final complement pathway should be preferred to treatments targeting a single upstream complement pathway. While targeting the complement pathway seems to be an attractive option in pMN patients, a preliminary trial of eculizumab – a monoclonal antibody that binds to C5 and prevents its cleavage – was unsuccessful (99). Other anti-complement therapies are being evaluated in pMN (100).

While anti-complement therapies have not yet been proven to be effective when administered alone, they could represent a complementary strategy when used simultaneously with other immunosuppressive drugs or prior to other immunosuppressive drugs. They may allow rapid containment of glomerular damage by blocking complement activation until the immunosuppressive treatment results in a sufficient decrease in circulating antibody levels. However, since part of the efficacy of monoclonal antibodies (e.g. rituximab) is based on complement-mediated cytotoxicity, anti-complement therapies could limit the effect of monoclonal antibodies if used simultaneously.

### 4.2 Elimination of Autoreactive B-Cells and Induction of Immune Tolerance

Chimeric antigen receptor (CAR) T cells is a technology based on T cells genetically engineered to express an artificial T cell receptor specific to an antigen of interest. CAR T cells are currently used in cancer immunotherapy (101) and are a promising therapeutic strategy to eliminate pathogenic B-cells in antibody-mediated autoimmune diseases (102). In a murine lupus model, CD19 CAR T cells persistently depleted CD19+ B-cells, eliminated autoantibody production, reduced the clinical manifestations of the disease and prolonged lifespan (103).

Chimeric autoantibody receptor T cells (CAAR T cells) are a modified version of CAR T cells. While CAR T cells recognize a specific surface antigen on the target cells, CAAR T cells contain domains of the antigen of interest. Thus, CAAR T cells recognize and bind to target autoantibodies expressed on autoreactive B-cells *via* the specific antigen and then destroy autoreactive cells. In a murine model of pemphigus vulgaris CAAR T cells expressing the target autoantigen – i.e. desmoglein 3 – specifically and efficiently eliminated desmoglein 3-specific autoreactive B-cells (104).

Another alternative is to induce immunotolerance as autoimmune diseases are characterized by a loss of self-tolerance. Since patients with pMN have a deficiency of regulatory T cells (13, 105, 106), the generation of CAR Tregs is a promising option to suppress autoimmune manifestations. CAR Tregs were effective in mouse models of encephalomyelitis and ulcerative colitis (107, 108). A second strategy to regain immune tolerance through the induction of Tregs is the use of nanoparticles with autoimmune disease-relevant peptides bound to major histocompatibility complex class II molecules. These nanoparticles may induce antigen-specific Tregs. These antigen-specific Tregs then promote the differentiation of B-cells into disease-suppressing regulatory B-cells, suppress autoantigen-loaded antigen-presenting cells and inhibit CD4+ and CD8+ T cells, leading to the resolution of autoimmune manifestations (109).

It would therefore be of interest to evaluate these new biotechnologies in the context of pMN refractory to conventional treatment.

### 4.3 Cytokine-Regulating Treatment

Cytokines may play a role in the pathogenesis of pMN. We have shown that high serum IL-17A levels were associated with poor prognosis in pMN, defined by more thromboembolic complications and more relapses of nephrotic syndrome. Rituximab treatment induced Th1 and regulatory T cell cytokines but did not impact Th17 cytokines (13). These data raise the question of additional maintenance therapy to block Th17-mediated inflammation. The blockade could be achieved by the use of anti-IL6 (e.g. siltuximab), anti-IL6 receptor (e.g. tocilizumab), anti-IL-17A (e.g. ixekizumab or secukinumab) or anti-IL-17 receptor (e.g. brodalumab) in combination with rituximab. Immunomodulators have the advantage of not inducing immunosuppression and therefore limit the adverse effects. These treatments could be of value for patients who relapse frequently. Further studies are needed to evaluate their effectiveness on prevention of relapses either alone or in combination with standard immunosuppressive treatment.

### 4.4 Other Immunosuppressive Treatments

Belimumab is a human IgG1-λ monoclonal antibody that inhibits B-cell activating factor (BAFF). In pMN patients it reduced anti-PLA2R1 antibody levels and proteinuria (110). When comparing this decrease in anti-PLA2R1 antibody levels with belimumab to that reported with rituximab in the GEMRITUX study, the decrease was faster in rituximab-treated patients than in belimumab-treated patients. It has been proposed that the faster effect of rituximab was related to immediate B-cell lysis, whereas the delayed effect of belimumab was related to progressive B-cell “exhaustion” secondary to BAFF binding and inhibition (111). Further studies are needed to assess the effectiveness of belimumab in pMN, alone or in combination with other therapies.

An ongoing clinical trial is also evaluating anti-CD38 antibody treatments to target plasma cells in pMN with anti-PLA2R1 antibodies (NCT0415440).

### 4.5 New Nephroprotective Therapies

New nephroprotective therapies have been developed in recent years. Sparsentan, a dual endothelin type A and angiotensin II type 1 receptor antagonist, has demonstrated superiority in reducing proteinuria over ibersartan in focal segmental glomerulosclerosis (FSGS) (112).

Preclinical studies suggest that inhibition of glomerular roundabout guidance receptor 2 (ROBO2)/slit guidance ligand 2 (SLIT2) signaling can stabilize podocyte adhesion and reduce proteinuria. A study is underway to evaluate the effectiveness of ROBO2/SLIT2 inhibition with the ROBO2 fusion protein PF-06730512 in patients with FSGS (113).

Sodium/glucose cotransporter 2 (SGLT2) inhibitors in combination with inhibitors of the renin angiotensin aldosterone system have been shown to be of value in preventing kidney failure in patients with chronic kidney disease (CKD) (114, 115).

Treatment with a steroidal mineralocorticoid receptor antagonist in patients with CKD reduces blood pressure and proteinuria (116). Finerenone, a selective non-steroidal mineralocorticoid receptor antagonist, in combination with inhibitors of the renin angiotensin aldosterone system, has been shown to be effective in reducing albuminuria in patients with diabetic nephropathy (117). Recently, finerenone has been shown to result in lower risks of CKD progression and cardiovascular events than placebo in patients with CKD and type 2 diabetes (118).

These treatments could also be of interest in pMN. Further studies are needed to evaluate their effectiveness in this indication.

## 5 Conclusion

Important advances have been made recently in the understanding of the pathophysiology and management of pMN. The KDIGO 2021 guidelines have drastically changed the approach of the disease management model from a homogeneous management of all patients to a personalized management based on the risk assessment of disease progression. Currently, rituximab is one of the first-line treatments for pMN with proven safety and efficacy. Recent advances in the understanding of the mechanisms behind the resistance to rituximab have made it possible to propose alternative and better adapted treatments in order to increase clinical remission rates and reduce the risk of relapse.

## Author Contributions

The original concept and design of the study was made by BS-P. MT performed a detailed literature search. MT, VE, MC, VB and BS-P drafted and revised the manuscript. All authors contributed to the article and approved the submitted version.

## Conflict of Interest

The authors declare that the research was conducted in the absence of any commercial or financial relationships that could be construed as a potential conflict of interest.

## Publisher’s Note

All claims expressed in this article are solely those of the authors and do not necessarily represent those of their affiliated organizations, or those of the publisher, the editors and the reviewers. Any product that may be evaluated in this article, or claim that may be made by its manufacturer, is not guaranteed or endorsed by the publisher.
